# How to write a grant proposal

**DOI:** 10.4103/0019-5413.30521

**Published:** 2007

**Authors:** Michael Zlowodzki, Anders Jönsson, Philip J Kregor, Mohit Bhandari

**Affiliations:** Division of Orthopedic Surgery, McMaster University, Hamilton, ON, Canada; * Association Internationale Pour l' Ostéosynthèse Dynamique, Nice, France; ** Department of Orthopedic Surgery, Vanderbilt University, Nashville, TN, USA

Academic success and promotion in medicine largely depends on the quality and quantity of received grants. Grant money brings prestige and notoriety to the writer and his institution. However, writing a grant proposal can be a challenging task especially for the inexperienced researcher. As research budgets are being reduced by many funding agencies and more researches are competing for it, it is becoming increasingly important to be able to write a grant proposal of high quality.

The purpose of this article is to give the reader guidance on how to organize a research proposal in order maximize chances to obtain the desired funding. Key aspects will be highlighted and practical tips emphasized. This article will primarily focus on writing a grant for a clinical study.

## GETTING STARTED

Good research starts with a good idea! Once you have identified a good idea, you need to clearly define the problem that needs to be addressed and formulate a research question. Subsequently you need to ask yourself if that question is already answered [Table 1]. A thorough literature review is therefore mandatory. If you have a truly good idea, you might find out that you are not the first one having it. You do not want to spend a lot of time and energy into a project only to find out later that there have been already 17 trials and a meta-analysis performed and your research question is answered.

**Table 1:** Checklist

What is the problem to be addressed? → Define the question
Is the question unanswered? → Literature research
Formulate hypothesis
Choose appropriate study design
Identify team and collaborators
Determine environmental and financial needs
Write study protocol

It is not only important to know how much was already published on that topic, but also what the quality of the current evidence is. Rarely in medicine does a question have a definitive answer. If you are trying to compare two interventions for a certain disease, after performing a thorough literature search, you have to ask yourself the following questions: 1) Are there already multiple case series published on that topic? If yes, then it might not be worth it to add another case series to the literature. However, that might be your chance for the first comparative study (cohort study or randomized controlled trial). 2) Are there already multiple comparative studies? If yes, are they cohort studies or randomized trials (RCT)? If there is no RCT maybe you should do one. 3) Are there already multiple RCTs published? If yes, what are the results and what is their sample size? Maybe they were underpowered? If yes you might consider doing a meta-analysis of the existing RCTs and subsequently a larger trial.

After you decided to perceive with your study proposal, you need to determine how many study subjects you need, how much money you need and who your collaborators will be. In order to be successful in obtaining a grant you will need convincing data, which might require several preliminary studies and you will need to prove to the granting agency that you are capable of performing the study the way you propose it. The purpose of the research plan is to describe what will be done, why it is important and how the study will be conducted.

## ELEMENTS OF A STUDY PROTOCOL

The key elements of the study protocol are the executive summary, specific aims, background and significance, preliminary results and research design and methods [Table 2]. The research design and the methodology used in the process of planning and conducting the project should be described in detail. Prior work relevant to the proposed project should be included. Also if a pilot study was conducted, the results should be included.

**Table 2:** Elements of a study protocol

Abstract (Executive summary) Research plan Specific aims Background and significance Preliminary results Research design and methods Budget and justification Resources and environment Timeline

### Abstract (Executive summary)

The abstract is an important part of a study protocol because it is the first page that a reviewer reads. Reviewers of granting agencies may make their opinion based on the abstract alone. It may be difficult to overcome a bad first impression and conversely there may be a lot to gain with a good first impression. The purpose of the abstract is to describe succinctly every key element of the proposed project. It is good to be complete but concise.

### Specific aims

The purpose of the specific study aims is to clearly describe what research question the investigators are trying to answer by conducting the study. What is the problem to be addressed? The investigators need to describe why the study is needed now. In detail, the hypothesis of the study and the primary and secondary goals should be stated. Typically, the study question should be formulated to include the following: 1) the population to be studied, 2) the intervention, 3) any comparison group to be studied (if relevant) and 4) the study outcomes. The study outcomes should be reported as the primary (main) outcome and any secondary outcomes.

### Background and significance

The purpose of the background and significance section is to lay out the rationale for the proposed research project and to summarize currently available data in the literature that is relevant to the project. If no systematic review or meta-analysis was done on the topic, you should do one. Describe the magnitude of the problem to be addressed. What is the patient population you are targeting? What is the incidence of the problem? Is the problem likely to increase in the future (e.g. geriatric fractures)? You need to describe the historic management of the problem and whether or not there is any consensus on the current management of the problem. Are there any uncertainties about the treatment that need to be resolved? If you hypothesize that intervention A is better than intervention B you need to designate your primary outcome parameter and have some baseline data for a sample size calculation. Depending on the project, you might want to survey surgeons for their treatment preferences. Also consider surveying patients to find out about what outcome they consider to be important. There might be some disagreements between the surgeons and patients perspectives.^1^ The purpose of the background and significance chapter is to justify the study you are proposing. Describe how the result of your study will benefit society. You need to convince the granting agencies that it is worth their money.

### Study design

In order to answer the question you need to choose an appropriate study design. The main clinical study designs are interventional studies, observational studies and diagnostic studies - some overlaps may exist [Table 3]. Which study design is most likely to answer the research question, which one is most feasible and which one gives the highest quality results? The choice of the study design has a significant implication on the magnitude of the required funding. Ethical considerations also need to be taken into account e.g. in some cases a certain study design might not be ethical. A clear description of the eligibility criteria (inclusion / exclusion) is essential. Also describe how outcomes will be measured during follow-up and what the follow-up schedule will be like (frequency and duration).

**Table 3:** Types of clinical study designs

Therapeutic study Randomized controlled trial Cohort study Case-control study Case series Observational study (prognostic) Prospective Retrospective Diagnostic study Testing previously developed diagnostic criteria Developing new diagnostic criteria Economic and decision analysis

### Sample size calculation

The sample size calculation is a crucial part of the study protocol. The required sample size has major implications on your required funding and the size of the team. Before you can calculate the sample size you need to designate the primary outcome. It is advantageous to choose an objective, reliable and highly validated outcome in order to limit bias. Ultimately, you should choose the clinically most important outcome that is feasible.

The sample size calculation is different depending on the type of the outcome; if you choose a categorical dichotomous outcome parameter (e.g. nonunion rate, infection rate) the sample size requirements are much higher than if you choose a continuous outcome like a score (e.g., SF-36, DASH, SMFA, pain score).^2^,^3^ In order to perform a sample size calculation for dichotomous outcomes, you must have an event rate (e.g., nonunion rate) for your gold standard treatment (e.g., treatment A) and you must hypothesize by how much treatment B is going to decrease or increase that event rate. For continuous outcomes you need to have a mean value for the gold standard treatment and hypothesize a difference for the alternative treatment. Using an alpha error rate of 0.05 (=accepting the probability of a false-positive result) and a beta error rate of 0.20 (=accepting the probability of a false-negative result), which corresponds to a power of 80% is a commonly accepted standard.

You can obtain baseline numbers either from a pilot study or reports in the literature. Ideally the “hypothesized” differences should be in the magnitude of what you consider clinically significant. You can calculate the sample size by hand^4^ or use one multiple tools to help with the sample size calculations^5^ [Table 4]. Be aware that the sample size calculation is based on assumptions; calculate the best-case and the worst-case scenario.

**Table 4:** Useful Books, Software and Websites

Otto O. Yang: Guide to Effective Grant Writing: How to Write a Successful NIH Grant Application, Springer, New York, 2005
Harvey Motulsky, Intuitive Biostatistics, Oxford University Press, New York, 1995
GraphPad InStat and StatMate (Statistical software)
http://www.r-project.org (Free statistical software)
http://www.stat.uiowa.edu/~rlenth/Power/ (Free power and sample size calculations)
http://www.niaid.nih.gov/ncn/grants/charts/checklists.htm#Planiict (NIH checklist)
http://www2.ejbjs.org/misc/instrux.shtml#levels (Levels of Evidence)

The justification of the estimated sample size should be presented as a separate section in a grant proposal. Investigators can present estimates of sample size varying across different mean differences between groups. Alternative approaches are to present the study power across varying sample sizes and mean differences or the estimated mean differences of the outcome parameter across varying study power.^4^

### Protecting against bias

Study results can be negatively affected by multiple types of bias, mainly selection bias and measurement bias. Investigators need to describe proposed methods for protecting against bias. The most powerful techniques for protecting against bias are 1) randomization, 2) concealment of randomization, 3) blinding and 4) the choice of an objective outcome measure.

If you are comparing the effect of multiple interventions on a specific outcome, the best method of protecting against selection bias is random treatment allocation. Randomization balances known and unknown prognostic factors between groups. Additionally, you can use techniques like blocking and stratification in order to avoid random imbalances in small randomized trials. If you do not allocate treatment options randomly, you should account for imbalances in prognostic factors between groups, by matching the patients to the different treatment groups based on the known prognostic factors upon enrollment in your study or if that is not possible, account for it in the data analysis. However, the only way to balance unknown prognostic factors is randomization.

Blinding is another important technique for protecting against bias. Investigators should blind whoever they can: the patient, the physician (not possible in surgical trials), the outcome assessor and the data analyst. Lastly it is helpful to choose an objective outcome measure like a validated functional outcome scale. If the outcome parameter is subjective (e.g., union/nonunion), you should consider to have an adjudication committee to assess the outcome.

## CONCLUSION

Grants are critical for success in academic medicine. The key to a good grant is a good idea and the ability to “sell” your idea to the reviewers of the granting agency. In order to “sell” your idea, good background research, the appropriate study design and a well thought out methodology are imperative. It is also important to recognize that research is a team effort. Convincing the grant reviewer of your expertise is crucial; choosing experienced team members therefore improves the chances to obtain the desired grant. A successful pilot study and preliminary studies that serve as a justification for your study proposal can prove feasibility to the grant reviewers and be therefore a persuasive factor. You should propose an appropriate budget and a realistic timeline; otherwise failure is almost certain. Lastly, you should tailor their grant application towards the granting agency's goals and use the requested format for their application as that might differ from agency to agency. Targeting multiple government and industry-funded agencies increases the chance of getting funded.
